# Identification and Characterization of a Cryptic Genomic Deletion-Insertion in *EYA1* Associated with Branchio-Otic Syndrome

**DOI:** 10.1155/2021/5524381

**Published:** 2021-04-05

**Authors:** Hao Zheng, Jun Xu, Yu Wang, Yun Lin, Qingqiang Hu, Xing Li, Jiusheng Chu, Changling Sun, Yongchuan Chai, Xiuhong Pang

**Affiliations:** ^1^Department of Otolaryngology-Head and Neck Surgery, Taizhou People's Hospital, The Fifth Affiliated Hospital of Nantong University & Clinical Hospital of Dalian Medical University, Taizhou, Jiangsu, China; ^2^Department of Clinical Medicine, Medical School of Nantong University, Nantong, Jiangsu, China; ^3^Department of Otolaryngology-Head and Neck Surgery, The Ninth People's Hospital, Shanghai Jiaotong University School of Medicine, Shanghai, China; ^4^Ear Institute, Shanghai Jiaotong University School of Medicine, Shanghai, China; ^5^Shanghai Key Laboratory of Translational Medicine on Ear and Nose Diseases, Shanghai, China; ^6^Department of Otolaryngology-Head and Neck Surgery, Affiliated Hospital of Jiangnan University, Wuxi, Jiangsu, China

## Abstract

Branchio-oto-renal spectrum disorder (BORSD) is characterized by hearing loss accompanied by ear malformations, branchial cysts, and fistulae, with (branchio-oto-renal syndrome (BORS)) or without renal abnormalities (BOS (branchio-otic syndrome)). As the most common causative gene for BORSD, dominant mutations in *EYA1* are responsible for approximately 40% of the cases. In a sporadic deaf patient diagnosed as BOS, we identified an apparent heterozygous genomic deletion spanning the first four coding exons and one 5′ noncoding exon of *EYA1* by targeted next-generation sequencing of 406 known deafness genes. Real-time PCR at multiple regions of *EYA1* confirmed the existence of this genomic deletion and extended its 5′ boundary beyond the 5′-UTR. Whole genome sequencing subsequently located the 5′ and 3′ breakpoints to 19268 bp upstream to the ATG initiation codon and 3180 bp downstream to exon 5. PCR amplification across the breakpoints in both the patient and his parents showed that the genomic alteration occurred *de novo*. Sanger sequencing of this PCR product revealed that it is in fact a GRCh38/hg38:chr8:g.71318554_71374171delinsTGCC genomic deletion-insertion. Our results showed that the genomic variant is responsible for the hearing loss associated with BOS and provided an example for deciphering such cryptic genomic alterations following pipelines of comprehensive exome/genome sequencing and designed verification.

## 1. Introduction

Branchio-oto-renal spectrum disorder (BORSD) characterized by malformations of the outer, middle, and inner ear associated with conductive, sensorineural, or mixed hearing loss, branchial cysts and fistulas, and renal abnormalities comprises branchio-oto-renal (BOR) syndrome (BOR1: #113650, BOR2: #610896) and branchio-otic syndrome (BOS) (BOS1: #602588, BOS3: #608389) [1, 2], two phenotypes that differ only by the presence or absence of renal abnormalities. BORSD affects about 1 in 40000 children including 2% of profoundly deaf children [3, 4]. Same to other dominant disorders, the offspring of BOR/BOS individuals are at a 50% risk of inheriting the pathogenic variant. Once the pathogenic variant has been identified in an affected family member, prenatal testing for a pregnancy or preimplantation genetic diagnosis (PGD) becomes possible [5, 6].

The known disease-causing genes for BOR/BOS are *EYA1* (#601653; located in 8q13.3), *SIX1* (#601205; located in 14q23.1), and *SIX5* (#600963; located in 19q13.32), in which *EYA1* is the most frequent gene responsible for about 40% of affected patients [7]. *EYA1*, the human homolog of the Drosophila eyes absent gene, acts as a protein phosphatase and transcriptional coactivator [8, 9]. Eya1 homozygous-deficient mice lack ears and kidneys, and Eya1 heterozygous-deficient mice present with phenotypes resembling BOR syndrome [10, 11]. The majority of disease-associated missense mutations cluster in the conserved C-terminal 271-residue Eya domain (ED) of *EYA1* (321-592 residues) [12]. Otherwise, the N-terminal domain of *EYA1* (1-320 residues) is poorly conserved and can attenuate the catalytic activity of Eya to achieve transactivation when bound to a DNA-binding protein [9, 13]. To date, more than 190 mutations in *EYA1* have been found to be associated with BOR/BOS, of which copy number variants (CNVs) account for about 17.1-20% (http://www.hgmd.cf.ac.uk/ac/all.php, last updated in April 2019) [7, 14, 15].

CNVs, DNA segments including deletions, duplications, and complex rearrangements which exceed 1 kb, are a major source of genome diversity in human populations [16, 17] and have been implicated in a variety of human diseases and cancers [18, 19]. Large CNVs, especially the heterozygous ones, however, were hard to be detected by the conventional mutation screening methods such as polymerase chain reaction (PCR) amplification and Sanger sequencing. With the wide application of various genome sequencing technologies, an increasing number of rare CNVs have been found to play a vital role in genetic etiology of hearing loss [20, 21]. But so far, there is no recognized pipeline for the detection of cryptic genomic alterations that are hard to detect by conventional methods.

In this study, we reported how multiple genomic sequencing methods including targeted NGS, real-time PCR, WGS, and Sanger sequencing were applied comprehensively to identify a heterozygous 55618 bp genomic deletion-insertion of *EYA1* gene in a sporadic patient with BOS. This may provide an example for deciphering such cryptic genomic alterations following pipelines of comprehensive exome/genome sequencing and designed verification.

## 2. Materials and Methods

### 2.1. Editorial Policies and Ethical Considerations

All subjects in this study gave written, informed consent to participate in this study. Identifying information will not be included in the manuscript unless the information is essential for scientific purposes. This study was approved by the Ethics Committee of Taizhou People's Hospital, the Fifth Affiliated Hospital of Nantong University, and was compliant with the Declaration of Helsinki.

### 2.2. Subjects and Clinical Examinations

**Figure 1:**
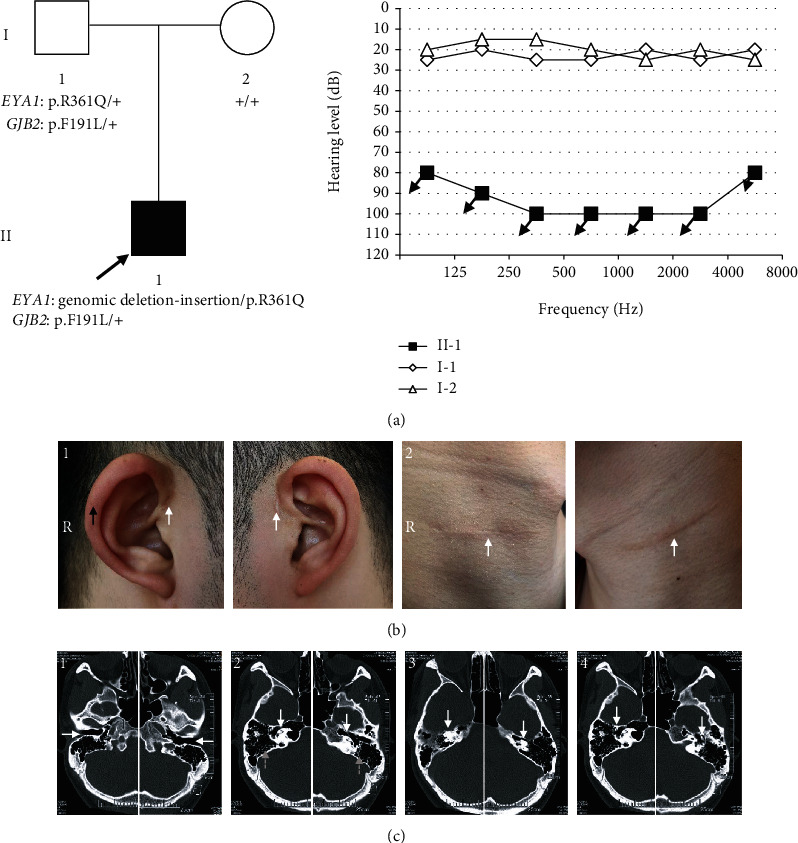
Pedigree, genotype, and phenotype characterization of the family. (a) Pedigree, genotype, and audiograms. Proband II-1 is pointed by the black arrow, and hearing loss is indicated by the black square. The audiograms showed profound sensorineural hearing loss in II-1 and normal hearing loss in his parents. (b) Right cup-shaped outer ear is showed by a black arrow in b1; two white arrows in b1 and b2 indicate bilateral surgical scars of preauricular fistula and cervical branchial cyst, respectively. (c) Findings in temporal HRCT: c1: bilateral lower external auditory canals; c2: white solid and grey dotted arrows indicate cochlear hypoplasia and overgasification of mastoid cells, respectively; c3: malformed semicircular canal; c4: deformed ossicular chain.

The proband II-1 and his parents (I-1 and I-2) were recruited by the Department of Otolaryngology—Head and Neck Surgery, Taizhou People's Hospital, Jiangsu Province (Figure 1(a)). Comprehensive clinical history was taken, and a detailed physical examination was performed in all subjects with special attentions to audiological, branchial, renal, olfactory, cardiac, ophthalmologic, skeletal, mental, intestinal, and dermatologic abnormalities. The hearing loss was confirmed by otoscopy, pure-tone audiometry (PTA), immittance, distortion product otoacoustic emission (DPOAE), and auditory brainstem response (ABR). The malformation of the middle and inner ear was confirmed by high-resolution CT (HRCT) and magnetic resonance imaging (MRI). Renal abnormalities were excluded by ultrasound and renal function test. Phenotypes of BOS/BOR were evaluated by the diagnostic criteria described previously [7]. Major criteria are branchial anomalies, deafness, preauricular pits, and renal anomalies. Minor criteria are external ear anomalies, middle ear anomalies, inner ear anomalies, preauricular tags, and others, including facial asymmetry and palate abnormalities.

### 2.3. Targeted NGS of 406 Deafness Genes

Genomic DNA was extracted from the whole blood using the Blood DNA Kit (TIANGEN BIOTECH, Beijing, China). Sequencing of all 406 deafness-related genes was completed by targeted next-generation sequencing (NGS) using the MyGenotics gene enrichment system (Panel1-V4, MyGenotics, Boston, MA, USA) and the Illumina HiSeq 2000 sequencer (Illumina, San Diego, CA, USA) as previously described (Supplementary Table S1) [22]. The reads were aligned to HG19 using the BWA software, and the variants were called using the Genome Analysis Toolkit (GATK), both with the default parameters. The copy numbers of related genes were obtained through CapCNV analysis followed by CNVkit protocol (https://cnvkit.readthedocs.io/en/stable/pipeline.html). SNVs and indels were presented using Variant Call Format (VCF) version 4.1 and annotated using the ANNOVAR software. Data analysis and bioinformatics processing were performed as previously described [22].

Possible pathogenic effect of the missense mutations was evaluated by computational tools including CADD, Exome Variant Server, gnomAD, MutationTaster, PolyPhen-2, 1000 genomes, PhastCons, Phylop, PROVEAN (cut-off score <–2.5), and SIFT (cut-off score < 0.05).

### 2.4. Real-Time PCR

**Figure 2:**
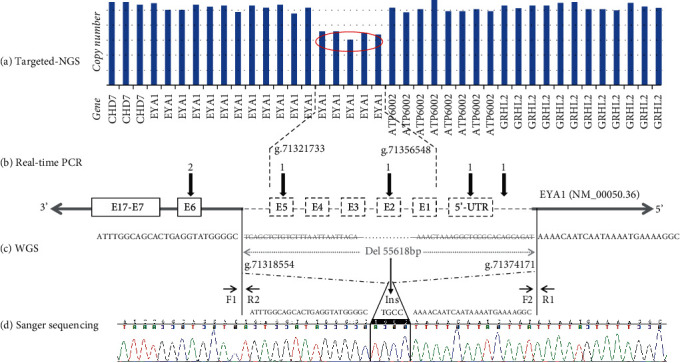
The process of identifying CNV. (a) The red oval indicates the region of deletion detected by targeted NGS. (b) Results of real-time PCR: 1 and 2 indicate copy number; black arrows: primer position; E: exon; left grey arrow: the 3′-end; right grey arrow: the 5′-end. (c) Grey words: region of deletion; F and R: forward and reverse primer; Ins: insertion. (d) Sequence diagram of 244 bp PCR product. 4 bases between the two black vertical lines were additional insertion.

To verify the existence and explore approximate breakpoint position of the suspected heterozygous genomic deletion related to exons 1-5 in *EYA1* found by targeted NGS of 406 deafness genes (Figure 2(a)), primers were designed in its upstream, middle, and downstream regions, including 5′-end upstream region, noncoding 5′-UTR, exon 2, exon 5, and exon 6 (Figure 2(b)). The primers were designed by the PRIME3 software online (http://frodo.wi.mit.edu/cgi-bin/primer3/primer3_www.cgi). Real-time PCR was performed in the proband, his unaffected parents, and a normal-hearing control on the 7300 Real-Time PCR System (Applied Biosystems) using SYBR® Premix Ex Taq™ (Takara Bio Company). Each reaction was repeated three times and the average Ct was recorded [21].

### 2.5. Whole Genome Sequencing

To judge whether the heterozygous deletion involved upstream genes or not and search for the exact breakpoint position of 5′- and 3′-end, whole genome sequencing was selected (Figure 2(c)). Paired-end DNA libraries were prepared according to the manufacturer's instructions (Illumina TruSeq Library Construction). DNA libraries were sequenced on Illumina HiSeq X according to the manufacturer's instructions for paired-end 150 bp reads. The average sequencing depth ranged from 31.35 to 57.77, and 90.1% to 99.2% of whole genome were covered at least 20. Reads (without barcode) were aligned to HG19 using SpeedSeq. Single nucleotide variants, insertions, deletions, and indels calling were performed using Genome Analysis Toolkit v2.1. Structure variants and copy number variants were analyzed in SpeedSeq. Annotations of single nucleotide variants, indels, structure variants, and copy number variants were performed with ANNOVAR [23].

### 2.6. Sanger Sequencing

To verify the results of WGS, a single PCR amplification was performed in the proband across the break junction (Figure 2(d)). The exact break junction and additional insertion were identified by sequencing of this PCR product. The same PCR amplification was used to detect the novel CNV in unaffected parents and a normal-hearing control (forward primer F1: 5′-ATCTGTGGCCCCCAAATACTTC-3′; reverse primer R1: 5′-AGGTCCTCTGCCCATTATTTGA-3′; PCR product size: 244 bp) (Figure 2(d)). In addition, a second PCR was performed to amplify across the 5′ breakpoint from the wild-type allele (forward primer F2: 5′-TTAGACCAGACACAAAAGCAACTCC-3′; reverse primer R1: 5′-AGGTCCTCTGCCCATTATTTGA-3′; PCR product size: 364 bp) (Figure 2(d)), and a third PCR was performed to amplify across the 3′ breakpoint from the wild-type allele (forward primer F1: 5′-ATCTGTGGCCCCCAAATACTTC-3′; reverse primer R2: 5′-AGAAAGGGATTTTCTAAAGCCATCA-3′; PCR product size: 559 bp) (Figure 2(d)). The CNV was determined as heterozygous if all PCR products were amplified and as homozygous if only the 244 bp but not the 364 bp and 559 bp products were amplified. No CNV was detected when only 364 bp and 559 bp were amplified. This CNV was subsequently screened in 400 ethnically matched normal controls (data not shown) and excluded benign CNV listing in the Database of Genomic Variants (http://dgv.tcag.ca/dgv/app/about?ref=).

## 3. Results

### 3.1. Clinical Characteristics

The sporadic patient II-1 was found with bilateral congenital profound sensorineural hearing loss (Figure 1(a)), right cup-shaped outer ear, bilateral old surgical scars of congenital preauricular fistula, and cervical branchial cysts (Figure 1(b)). Bilateral lower external auditory canals, enlarged middle ear cavity, overgasification of mastoid cells, malformed ossicular chain, cochlear hypoplasia in immature apical turn and absence of the middle turn, malformed semicircular canals, and abnormal internal auditory canals were found by temporal bone HRCT (Figure 1(c)). Renal and other abnormalities were excluded after a series of detailed clinical examinations.

### 3.2. Screening for All Known Deafness Genes by Targeted NGS

**Figure 3:**
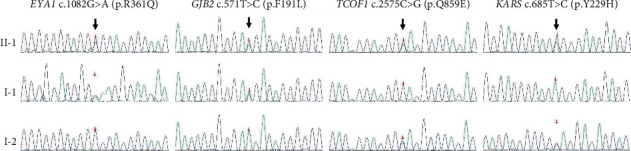
Sequencing diagram of 4 missense variants in patient II-1, unaffected parents I-1/I-2. Black arrow: changed base position.

Five heterozygous variants were submitted by targeted NGS of 406 known deafness genes in this patient: a genomic deletion spanning coding exon 2-6 in *EYA1* (GRCh38/h38: chr8: 71321733-71356548) (Figure 2(a)), c.1082G>A (p.Arg361Gln) in *EYA1* (NM_000503.6), c.571T>C (p.Phe191Leu) in *GJB2* (NM_004004.6), c.2575C>G (p.Gln859Glu) in *TCOF1* (NM_001135243), and c.685T>C (p.Tyr229His) in *KARS* (NM_001130089) (Figure 3). Possible pathogenic effect of the missense mutations was evaluated by computational tools (Table 1). Variants p.Arg361Gln (rs145219836) in *EYA1* and p.Phe191Leu (rs397516878) in *GJB2* were proved to be inherited from his unaffected father, while p.Gln859Glu (rs201043592) in *TCOF1* and p.Tyr229His (rs150529876) in *KARS* were from his unaffected mother (Figure 3 and Table 1).

### 3.3. Verify the Existence of the Deletion by Real-Time PCR

To verify if the genomic deletion found by targeted NGS existed or not, the DNA segments in 5′-upstream region, 5′-UTR, exon 2, exon 5, and exon 6 were detected quantitatively, and their copy numbers were 1, 1, 1, 1, and 2, respectively (Figure 2(b)). The genomic deletion was proved to exist and its 3′-end breakpoint located within exon 5-exon 6. But where the 5′-end breakpoint was and whether the deletion involved upstream genes or not need further exploration.

### 3.4. Breakpoints of the Deletion Identified by WGS

To explore where the 5′-end breakpoint was and whether the deletion involved upstream genes or not, WGS was selected. 55618 bp genomic deletion located in chromosome 8q13.3 was identified from g.71318554 to g.71374171 (19268 bp upstream to initiation codon ATG and 3180 bp downstream to exon 5), which involved 5′ upstream, noncoding 5′-UTR and exon 1, coding exons 2-5, intron 1-4, and partial intron 5 (Figure 2(c)).

### 3.5. True Genomic CNV Was Identified by Sanger Sequencing

**Figure 4:**
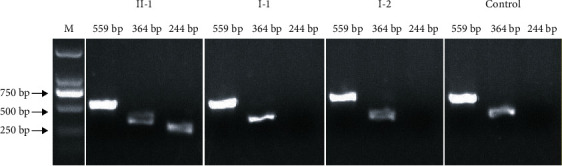
Electrophoresis gel figure of 244 bp, 364 bp, and 559 bp PCR product in patient II-1, unaffected parents I-1/I-2, and control. M: marker.

The breakpoints detected by WGS were verified exactly the same by amplification of the 244 bp product across 5′ and 3′ breakpoints. Notably, an additional 4 bp insertion not detected by WGS was identified by Sanger sequencing. So a novel deletion-insertion variant GRCh38/hg38:chr8:g.71318554_71374171delinsTGCC spanned 5′-UTR, exons 1 to 5, was identified in our study (Figure 2(d)). 559 bp and 364 bp products amplified successfully suggested that the deletion-insertion variant was a heterozygous one. Meanwhile, these three fragments (559 bp, 364 bp, and 244 bp) also amplified in unaffected parents and one normal-hearing control, only fragments of 559 bp and 364 bp but 244 bp amplified successfully in them (Figure 4).

Moreover, the novel deletion-insertion variant in *EYA1* was not found in 400 ethnically matched normal controls and was ruled out as a benign CNV listed in the Database of Genomic Variants (DGV, http://dgv.tcag.ca/dgv/app/about?ref=).

## 4. Discussion

Hearing loss is one of the major disabilities worldwide, which is often induced by loss of sensory hair cells in the inner ear cochlea [24–28]. Hearing loss could be caused by genetic factors, aging, chronic cochlear infections, infectious diseases, ototoxic drugs, and noise exposure [29–37]; and genetic factors account for more than 60% of hearing loss. According to phenotypes of BOS/BOR evaluated by the diagnostic criteria described previously [7], BOS was diagnosed in our sporadic patient with three major criteria of branchial anomaly, deafness, and preauricular pits and three minor criteria of external, middle, and inner ear anomalies. In contrast to the deformities of the ear reported in a previous study which showed atresia or stenosis in the external auditory canal, reduction in size of the middle ear space and hypoplastic mastoid cells [2], enlarged middle ear cavity, and overgasification of mastoid cells were discovered in our study (Figure 1(c)). In addition, the lower position of bilateral external auditory canals was first reported. High heterogeneity of phenotypes in BOR spectrum disease was further confirmed by our findings [7].

A heterozygous missense variant p.Arg361Gln and a genomic CNV GRCh38/hg38:chr8:g.71318554_71374171delinsTGCC in *EYA1* were identified simultaneously in our study. The former was suggested to be a nonpathogenic variant due to inheriting from his phenotypically normal father and benign predicted result of PolyPhen-2, PROVEAN, and SIFT (Figures 1 and 3, Table 1). The latter, a novel genomic deletion-insertion variant spanned 5′-UTR, exons 1 to 5, involving 5′-UTR and N-terminal domain, was very likely to be a pathogenic mutation, due to it being not found in his phenotypically normal parents (Figure 4) and 400 ethnically matched normal controls, and was ruled out as a benign CNV listed in the DGV. *De novo* mutation was proved by the parental origin of variants p.Arg361Gln in *EYA1*, p.Phe191Leu in *GJB2*, p.Gln859Glu (rs201043592) in *TCOF1*, and p.Tyr229His in *KARS* (Figures 1 and 3, Table 1). This result further proved previous opinion about high *de novo* rate in *EYA1* [38].

To date, two pathogenic mutations c.241C>T (p.Gln81Ter) and c.229C>T (p.Arg77Ter) (NM_000503.6) involving exons 1-5 are reported in previous study. They are predicted to be pathogenic due to loss of function from truncated or absent protein (https://www.ncbi.nlm.nih.gov/clinvar/variation). These two pathogenic mutations were included in the range of the novel genomic CNV discovered in our study. Haploinsufficiency seems to be the most likely explanation for BOR-related phenotypes in cases with nonsense and large deletions leading to similar disease phenotypes [39, 40]. In addition, the presence of the N-terminal domain significantly attenuates the phosphatase activity of Eya [9]. It has been proposed that the ED domain of Eya acts as an autoinhibitor of the transactivation potential of the N-terminal domain [41]. A decreased ability in binding to special proteins of N-terminal domain caused by haploinsufficiency led to a decline in transactivation of EYA1, which was very likely the pathogenic mechanism of the genomic deletion-insertion mutation identified in our study [9, 13].

So far, no specialized detection scheme was considered reasonable for rapid and economical detection of cryptic genomic CNVs. In our study, by applying multiple genomic testing methods step by step to identify a potential heterozygous genomic CNV (Figure 2), a rational pipeline for detecting cryptic genomic CNVs appeared to have been successfully established. Since most of the pathogenic mutations are located in the coding region and its flanking sequences and the cost of targeted NGS and WES is relatively lower than that of WGS, researchers often choose one of them to search for pathogenic mutations first. A potential heterozygous genomic deletion involving exon 1-exon 5 of *EYA1* was reported by targeted NGS (Figure 2(a)). To verify whether the deletion existed and the range of 5′- and 3′-end breakpoints, real-time PCR was considered as the preferred method for quantification [21]. Unfortunately, while confirming the presence of the deletion, 5′-end breakpoint region could not be evaluated by real-time PCR (Figure 2(b)). Considering economy, accuracy, time, and manpower saving, WGS was selected to detect the exact breakpoints. Encouragingly, the position of 5′- and 3′-end breakpoints detected by WGS was proved perfectly accurate by Sanger sequencing (Figures 2(c) and 2(d)). However, if there was no verification by Sanger sequencing, the “TGCC” insertion that WGS failed to detect would have been missed (Figure 2(d)). In the process of detection, we discovered that all molecular detection methods applied above had their own special advantages and disadvantages; reasonable and comprehensive application of them was of great significance for the efficient detection of cryptic heterozygous genomic CNV. Targeted NGS and WES only detected exons and their flanking sequences, but not noncoding regions, so they can only provide clues for the possible existence of CNVs. Fortunately, due to no restriction on the region of primers for quantitative detection, real-time PCR can well make up for the defects of targeted NGS and WES and can confirm the existence of CNV and the range of its breakpoints no matter in coding or noncoding regions. When the region of CNV breakpoints cannot be determined, WGS can be selected to find the breakpoints accurately in the genomic level. However, given that the read length of WGS is about 150-200 bp, short fragment may be missed for complex CNVs, such as deletion-insertion. As the most accurate sequencing technology, Sanger sequencing can detect each base missed by WGS in targeted amplified region and thus dig out the true genomic CNVs. So we suggested that the reasonable application of the above sequencing methods step by step can be used as a pipeline for detection of cryptic genomic CNVs in our future work.

According to the parental origin and pathogenic prediction of computational tools, missense p.Arg361Gln in *EYA1*, p.Gln859Glu (rs201043592) in *TCOF1*, and p.Tyr229His in *KARS* were considered as benign variants (Figures 1 and 3, Table 1). Although p.Phe191Leu in *GJB2* was listed as uncertain significance in ClinVar (https://www.ncbi.nlm.nih.gov/clinvar), it was regarded as a recessive inherited pathogenic one in our daily counseling work according to functional study [42]. Unfortunately, biallelic pathogenic mutations c.235delC and c.176_191del of *GJB2* were identified in his girlfriend with nonsyndromic profound hearing loss. According to the law of autosomal recessive inheritance, the offspring of the proband and his girlfriend is at a 50% risk of inheriting biallelic pathogenic variants of *GJB2*. To *EYA1*, also 50% of their offspring should be affected with BOR spectrum diseases in an autosomal dominant way. Therefore, it was of great significance to identify the genetic pathogenic factors for them, which provides a theoretical basis for prenatal diagnosis or PGD to get healthy offspring. Given that the chance of conceiving healthy offspring naturally was only 25%, we suggested that PGD technology was their best choice.

## 5. Conclusion

In conclusion, a novel heterozygous *de novo* genomic deletion-insertion in *EYA1*, GRCh38/hg38:chr8:.71318554_71374171delinsTGCC, was very likely the pathogenic cause for the patient with BOS due to a decline in transactivation of EYA1 resulting from haploinsufficiency. Through genetic counseling, the disease from *EYA1* and *GJB2* in the offspring of the patient can be avoided in the process of subsequent reproduction. Our results provided an example for deciphering such cryptic genomic alterations following pipelines of comprehensive exome/genome sequencing and designed verification.

## Figures and Tables

**Table 1:** Pathogenic prediction by computational tools.

Gene	Mutation	CADD-phred	Exome Variant Server	gnomAD	1000G	MutationTaster	PhastCons scores^∗^	Phylop score^†^	PolyPhen-2 (HumVar score)	PROVEAN (score)^‡^	SIFT (score)^§^	Origin
*EYA1*	p.Arg361Gln c.1082G>A rs145219836	26.7	Not present	0.0005	0.0002	Disease causing (0.999)	0.995	6.162	Benign (0.123)	Neutral (-1.86)	Tolerated (0.114)	I-1
*GJB2*	p.Phe191Leu c.571T>C rs397516878	27.0	Not present	0.000149	0.0002	Disease causing (0.999)	1	5.159	Possibly damaging (1)	Deleterious (-5.72)	Damaging (0.006)	I-1
*TCOF1*	p.Q859E c.2575C>G rs201043592	11.16	Not present	0.000107	0.0005	Polymorphism	0.139	1.479	Benign (0.301)	Neutral (-1.56)	Damaging (0.046)	I-2
*KARS*	p.Y229H c.685T>C rs150529876	15.34	Not present	0.001161	0.0032	Polymorphism	0.006	-0.014	Benign (0.275)	Neutral (0.36)	Tolerated (0.593)	I-2

Note: ^∗^The values vary between 0 and 1; the closer the value is to 1, the more probable the nucleotide is conserved. ^†^The values between -14 and +6, sites predicted to be conserved are assigned positive scores, while sites predicted to be fast evolving are assigned negative scores. ^‡^Negative and positive scores indicate deleterious and neutral, respectively, with cut-off score set at -2.5. ^§^The value ranges from 0 (deleterious) to 1 (neutral) with cut-off score set at 0.05.

## Data Availability

All the sequencing data supporting the conclusions of the study can be obtained by contacting Xiuhong Pang via email (pxhzxy@163.com).

## Supplementary Materials

Supplementary Materials Supplementary Table S1: 406 causative genes for deafness were screened by targeted NGS.
